# 1,4-Bis[(2-pyridyl­eth­yl)imino­meth­yl]benzene

**DOI:** 10.1107/S1600536811009809

**Published:** 2011-03-19

**Authors:** Haleden Chiririwa, John R. Moss, Hong Su, Denver Hendricks, Reinout Meijboom

**Affiliations:** a Department of Chemistry, University of Cape Town, Private Bag, Rondebosch 7707, South Africa; b Division of Medical Biochemistry, Faculty of Health Sciences, Private Bag X3, Observatory 7935, South Africa; c Department of Chemistry University of Johannesburg, PO Box 524, Auckland Park, Johannesburg 2006, South Africa.

## Abstract

In the title compound, C_22_H_22_N_4_, the centroid of the benzene ring is located on an inversion centre. The dihedral angle between the benzene and pyridine rings is 10.94 (5)°. The crystal structure displays weak inter­molecular C—H⋯N hydrogen bonding and C—H⋯π inter­actions.

## Related literature

For related compounds, see: Chakraborty *et al.* (1999 ▶); Haga *et al.* (1985 ▶). 
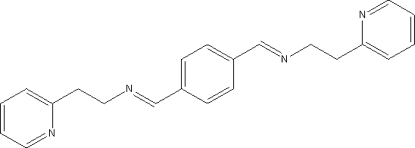


## Experimental

### 

#### Crystal data

C_22_H_22_N_4_
*M* *_r_* = 342.44
Monoclinic, 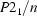

*a* = 6.0078 (6) Å
*b* = 26.023 (3) Å
*c* = 6.1319 (7) Å
β = 106.009 (2)°
*V* = 921.47 (17) Å^3^
*Z* = 2
Mo *K*α radiation
μ = 0.08 mm^−1^
*T* = 173 K
0.26 × 0.24 × 0.17 mm

#### Data collection

Bruker Kappa DUO APEXII diffractometer
11941 measured reflections
2288 independent reflections
1945 reflections with *I* > 2σ(*I*)
*R*_int_ = 0.024

#### Refinement

*R*[*F* ^2^ > 2σ(*F* ^2^)] = 0.040
*wR*(*F* ^2^) = 0.110
*S* = 1.06
2288 reflections
118 parameters
H-atom parameters constrained
Δρ_max_ = 0.28 e Å^−3^
Δρ_min_ = −0.20 e Å^−3^

### 

Data collection: *APEX2* (Bruker, 2006 ▶); cell refinement: *SAINT* (Bruker, 2006 ▶); data reduction: *SAINT*; program(s) used to solve structure: *SHELXS97* (Sheldrick, 2008 ▶); program(s) used to refine structure: *SHELXL97* (Sheldrick, 2008 ▶); molecular graphics: *X-SEED* (Barbour, 2001 ▶); software used to prepare material for publication: *publCIF* (Westrip, 2010 ▶).

## Supplementary Material

Crystal structure: contains datablocks I, global. DOI: 10.1107/S1600536811009809/go2006sup1.cif

Structure factors: contains datablocks I. DOI: 10.1107/S1600536811009809/go2006Isup2.hkl

Additional supplementary materials:  crystallographic information; 3D view; checkCIF report

## Figures and Tables

**Table 1:** Hydrogen-bond geometry (Å, °) *Cg*1 and *Cg*2 are the centroids of the C1–C5/N1 and C9–C11/C9′–C11′ rings, respectively.

*D*—H⋯*A*	*D*—H	H⋯*A*	*D*⋯*A*	*D*—H⋯*A*
C3—H3⋯N1^i^	0.95	2.74	3.544 (3)	143 (3)
C4—H4⋯N2^i^	0.95	2.69	3.593 (2)	159 (4)
C7—H7*A*⋯N1^ii^	0.99	2.87	3.847 (2)	171 (5)
C2—H2⋯*Cg*1^iii^	0.95	2.88	3.826 (4)	172 (5)
C6—H6*A*⋯*Cg*2^iv^	0.99	2.90	3.508 (3)	120 (2)

Symmetry codes: (i) 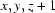; (ii) 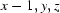; (iii) 
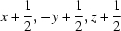
; (iv) 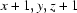.

**Fig. 1.**
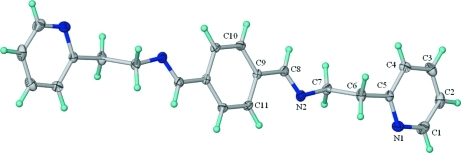
Molecular structure of titled compound showing the atomic numbering scheme. All non-hydrogen atoms were presented with ellipsoidal model with probability level 40%. Half of the molecule without atomic labels was generated via centre of symmetry (symmetry code: -x, -y, -z).

**Fig. 2.**
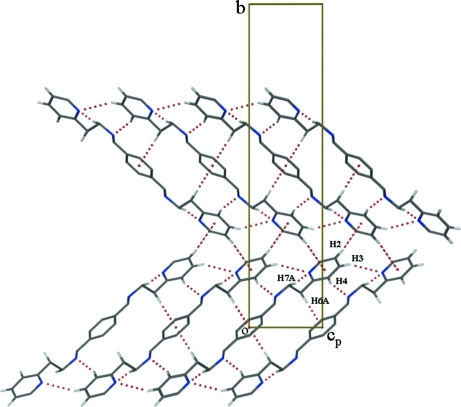
Projection viewed along [100] showing 3-D herringbone packing. Only the hydrogen atoms that invloved in C—H···N and C—H···π intermolecular interactions (see the list in Table 1) are shown and labelled. The red dotted lines represent the weak interactions.

## supplementary crystallographic information

### Comment

This work originates from our interest in developing a new class of tetradentate ligands. To the best of our knowledge, this work demonstrates the first example of neutral pyridinyldimine-based bridging ligand. The title compound might be expected to behave as a tetradentate chelating agent, in which both of the N atoms from the imine might coordinate, along with the two pyridinyl N atoms. Chakraborty *et al.* (1999) reported coordination of similar ligands to ruthenium whilst Haga and Koizumi (1985) reported their coordination to molybdenum. The structure of the title compound crystallized in space group *P*2_1_/*n* with *Z* = 2. The molecule, shown in Fig. 1, has a center of inversion at the centroid of the benzene ring and was located in special positions at Wyckoff positon *a*. The conformation of the molecule is best described by the dihedral angle of the central ring and pyridyl ring of 10.94 (5)°. The structure is stabilized by weak hydrogen bonds of the type C—H···N and C—H···π, the metrics of which are given in Table 1. The C—H···N intermolecular interactions, as well as C6—H6A···Ring 1 (of C10—C9—C11—C10'-C9'-C11'), connect the parallel neighbouring molecules into 2-dimentional layers. And these layers are then linked along the *b* axis into 3-dimentional herringbone packing *via* C2—H2···Ring 2 (of C1—C2—C3—C4—C5—N1) interactions, as shown in Fig.2.

### Experimental

The title compound was synthesized as follows: a solution of benzene 1,4-dicarboxaldehyde (0.50 g, 3.73 mmol) in methanol (10 ml) was added dropwise to a stirred solution of 2-(pyridin-2-yl)ethanamine(0.91 g, 7.42 mmol) in methanol (10 ml). The mixture was stirred at room temperature for *ca* 16 h. The precipitate was filtered off and washed with diethylether and dried under vacuum for 4 h affording a fine shiny white powder in 85% yield. M.p.: does not melt below 260 °C. Recrystallization by slow diffusion of Et_2_O into a concentrated CH_2_Cl_2_ of the solution gave colorless crystals suitable for X-ray structure analysis.

### Refinement

All non-hydrogen atoms were refined anisotropically and all hydrogen atoms were placed in idealized positions and refined with a riding model with *U*_iso_ set at 1.2 or 1.5 times *U*_eq_ of their parent atoms and fixed C—H bond lengths.

### Crystal data

**Table:**

C_22_H_22_N_4_	*F*(000) = 364
*M**_r_* = 342.44	*D*_x_ = 1.234 Mg m^−^^3^
Monoclinic, *P*2_1_/*n*	Mo *K*α radiation, λ = 0.71073 Å
Hall symbol: -P 2yn	Cell parameters from 11941 reflections
*a* = 6.0078 (6) Å	θ = 3.1–28.3°
*b* = 26.023 (3) Å	µ = 0.08 mm^−^^1^
*c* = 6.1319 (7) Å	*T* = 173 K
β = 106.009 (2)°	Plate, colourless
*V* = 921.47 (17) Å^3^	0.26 × 0.24 × 0.17 mm
*Z* = 2	

### Data collection

**Table:**

Bruker Kappa DUO APEXII diffractometer	1945 reflections with *I* > 2σ(*I*)
Radiation source: fine-focus sealed tube	*R*_int_ = 0.024
graphite	θ_max_ = 28.3°, θ_min_ = 3.1°
0.5° φ scans and ω	*h* = −8→8
11941 measured reflections	*k* = −34→33
2288 independent reflections	*l* = −8→8

### Refinement

**Table:**

Refinement on *F*^2^	Primary atom site location: structure-invariant direct methods
Least-squares matrix: full	Secondary atom site location: difference Fourier map
*R*[*F*^2^ > 2σ(*F*^2^)] = 0.040	Hydrogen site location: inferred from neighbouring sites
*wR*(*F*^2^) = 0.110	H-atom parameters constrained
*S* = 1.06	*w* = 1/[σ^2^(*F*_o_^2^) + (0.0506*P*)^2^ + 0.2363*P*] where *P* = (*F*_o_^2^ + 2*F*_c_^2^)/3
2288 reflections	(Δ/σ)_max_ < 0.001
118 parameters	Δρ_max_ = 0.28 e Å^−^^3^
0 restraints	Δρ_min_ = −0.20 e Å^−^^3^

### Special details

**Table:**

Experimental. Data for (I):IR (KBr): 1610 cm^-1^ (C=N, imine) ^1^HNMR:(CDCl_3_) δ_H_ 8.55(ddd, 2H, *J* =0.8 Hz, *J* = 1.7 Hz, J = 4.8 Hz) 8.21 (t, 2H, *J* = 1.3 Hz) 7.69 (s, 2H) 7.55 (dt, 2H, *J* = 1.9 Hz, *J* = 7.7 Hz) 7.11 (m, 2H) 4.03 (dt, 8H, *J* = 1.2 Hz, *J* = 7.2 Hz) 3.19(t, 4H, *J* = 7.2 Hz); ^13^CNMR: (400 MHz, CDCl_3_) δ 161.05, 159.45, 149.37, 138.88, 136.13, 128.21, 123.67, 121.24, 61.18, 39.61; Analysis calculated for C_22_H_22_N_4_:*C*, 77.16%; H, 6.48%; N, 16.36%; Found: C, 77.19%; H, 6.22%; N, 16.52%; EI—MS: *m/z* 249.90[*M* – C_7_H_6_N]^+^.Half sphere of data collected using *SAINT* strategy (Bruker, 2006). Crystal to detector distance = 50 mm; combination of φ and ω scans of 0.5°, 40 s per °, 2 iterations.
Geometry. All e.s.d.'s (except the e.s.d. in the dihedral angle between two l.s. planes) are estimated using the full covariance matrix. The cell e.s.d.'s are taken into account individually in the estimation of e.s.d.'s in distances, angles and torsion angles; correlations between e.s.d.'s in cell parameters are only used when they are defined by crystal symmetry. An approximate (isotropic) treatment of cell e.s.d.'s is used for estimating e.s.d.'s involving l.s. planes.
Refinement. Refinement of *F*^2^ against ALL reflections. The weighted *R*-factor *wR* and goodness of fit *S* are based on *F*^2^, conventional *R*-factors *R* are based on *F*, with *F* set to zero for negative *F*^2^. The threshold expression of *F*^2^ > σ(*F*^2^) is used only for calculating *R*-factors(gt) *etc*. and is not relevant to the choice of reflections for refinement. *R*-factors based on *F*^2^ are statistically about twice as large as those based on *F*, and *R*- factors based on ALL data will be even larger.

### Fractional atomic coordinates and isotropic or equivalent isotropic displacement parameters (Å^2^)

**Table:**

	*x*	*y*	*z*	*U*_iso_*/*U*_eq_	
N1	1.01974 (18)	0.16910 (4)	0.82099 (17)	0.0365 (3)	
C1	1.1434 (2)	0.20288 (5)	0.9711 (2)	0.0431 (3)	
H1	1.2675	0.2201	0.9339	0.052*	
N2	0.40565 (16)	0.09448 (4)	0.35620 (15)	0.0282 (2)	
C2	1.1022 (2)	0.21422 (5)	1.1750 (2)	0.0406 (3)	
H2	1.1936	0.2389	1.2748	0.049*	
C3	0.9248 (2)	0.18885 (5)	1.2306 (2)	0.0381 (3)	
H3	0.8928	0.1952	1.3715	0.046*	
C4	0.7937 (2)	0.15394 (4)	1.07884 (19)	0.0312 (3)	
H4	0.6689	0.1363	1.1130	0.037*	
C5	0.84667 (18)	0.14496 (4)	0.87569 (18)	0.0252 (2)	
C6	0.7121 (2)	0.10708 (4)	0.7047 (2)	0.0327 (3)	
H6A	0.6662	0.0778	0.7860	0.039*	
H6B	0.8134	0.0936	0.6152	0.039*	
C7	0.49617 (19)	0.13015 (4)	0.54368 (19)	0.0283 (2)	
H7A	0.3778	0.1365	0.6254	0.034*	
H7B	0.5346	0.1634	0.4844	0.034*	
C8	0.21489 (18)	0.07288 (4)	0.34628 (17)	0.0251 (2)	
H8	0.1384	0.0810	0.4584	0.030*	
C9	0.10603 (17)	0.03556 (4)	0.16772 (17)	0.0231 (2)	
C10	−0.10072 (18)	0.01157 (4)	0.17194 (18)	0.0259 (2)	
H10	−0.1702	0.0196	0.2894	0.031*	
C11	0.20546 (18)	0.02380 (4)	−0.00674 (18)	0.0254 (2)	
H11	0.3453	0.0401	−0.0122	0.030*	

### Atomic displacement parameters (Å^2^)

**Table:**

	*U*^11^	*U*^22^	*U*^33^	*U*^12^	*U*^13^	*U*^23^
N1	0.0364 (6)	0.0409 (6)	0.0340 (5)	−0.0050 (4)	0.0129 (4)	0.0008 (4)
C1	0.0345 (6)	0.0402 (7)	0.0532 (8)	−0.0114 (5)	0.0095 (6)	0.0025 (6)
N2	0.0260 (5)	0.0295 (5)	0.0272 (5)	−0.0033 (4)	0.0044 (4)	−0.0064 (4)
C2	0.0428 (7)	0.0273 (6)	0.0400 (7)	−0.0030 (5)	−0.0081 (5)	−0.0037 (5)
C3	0.0552 (8)	0.0312 (6)	0.0266 (6)	0.0043 (5)	0.0089 (5)	−0.0037 (5)
C4	0.0354 (6)	0.0288 (5)	0.0317 (6)	−0.0010 (5)	0.0131 (5)	−0.0008 (4)
C5	0.0249 (5)	0.0240 (5)	0.0242 (5)	0.0031 (4)	0.0025 (4)	0.0002 (4)
C6	0.0342 (6)	0.0275 (6)	0.0311 (6)	0.0025 (5)	0.0000 (5)	−0.0061 (4)
C7	0.0257 (5)	0.0278 (5)	0.0296 (5)	−0.0019 (4)	0.0045 (4)	−0.0074 (4)
C8	0.0244 (5)	0.0252 (5)	0.0250 (5)	0.0007 (4)	0.0056 (4)	−0.0027 (4)
C9	0.0219 (5)	0.0217 (5)	0.0239 (5)	0.0004 (4)	0.0034 (4)	−0.0009 (4)
C10	0.0252 (5)	0.0284 (5)	0.0252 (5)	−0.0010 (4)	0.0088 (4)	−0.0026 (4)
C11	0.0214 (5)	0.0262 (5)	0.0289 (5)	−0.0030 (4)	0.0073 (4)	−0.0014 (4)

### Geometric parameters (Å, °)

**Table:**

N1—C5	1.3344 (15)	C6—C7	1.5204 (16)
N1—C1	1.3395 (17)	C6—H6A	0.9900
C1—C2	1.372 (2)	C6—H6B	0.9900
C1—H1	0.9500	C7—H7A	0.9900
N2—C8	1.2628 (14)	C7—H7B	0.9900
N2—C7	1.4609 (13)	C8—C9	1.4748 (14)
C2—C3	1.3739 (19)	C8—H8	0.9500
C2—H2	0.9500	C9—C11	1.3954 (14)
C3—C4	1.3811 (17)	C9—C10	1.3968 (14)
C3—H3	0.9500	C10—C11^i^	1.3848 (14)
C4—C5	1.3880 (15)	C10—H10	0.9500
C4—H4	0.9500	C11—C10^i^	1.3848 (14)
C5—C6	1.5023 (15)	C11—H11	0.9500
			
C5—N1—C1	117.33 (10)	C7—C6—H6B	109.0
N1—C1—C2	124.19 (12)	H6A—C6—H6B	107.8
N1—C1—H1	117.9	N2—C7—C6	109.03 (9)
C2—C1—H1	117.9	N2—C7—H7A	109.9
C8—N2—C7	117.17 (9)	C6—C7—H7A	109.9
C1—C2—C3	118.02 (11)	N2—C7—H7B	109.9
C1—C2—H2	121.0	C6—C7—H7B	109.9
C3—C2—H2	121.0	H7A—C7—H7B	108.3
C2—C3—C4	119.10 (11)	N2—C8—C9	122.66 (9)
C2—C3—H3	120.4	N2—C8—H8	118.7
C4—C3—H3	120.4	C9—C8—H8	118.7
C3—C4—C5	119.07 (11)	C11—C9—C10	119.15 (9)
C3—C4—H4	120.5	C11—C9—C8	121.17 (9)
C5—C4—H4	120.5	C10—C9—C8	119.68 (9)
N1—C5—C4	122.27 (10)	C11^i^—C10—C9	120.70 (9)
N1—C5—C6	116.12 (10)	C11^i^—C10—H10	119.6
C4—C5—C6	121.60 (10)	C9—C10—H10	119.6
C5—C6—C7	113.13 (9)	C10^i^—C11—C9	120.15 (9)
C5—C6—H6A	109.0	C10^i^—C11—H11	119.9
C7—C6—H6A	109.0	C9—C11—H11	119.9
C5—C6—H6B	109.0		
			
C5—N1—C1—C2	−0.4 (2)	C8—N2—C7—C6	111.41 (11)
N1—C1—C2—C3	0.9 (2)	C5—C6—C7—N2	167.84 (9)
C1—C2—C3—C4	−1.10 (19)	C7—N2—C8—C9	−179.14 (9)
C2—C3—C4—C5	0.83 (18)	N2—C8—C9—C11	−2.76 (16)
C1—N1—C5—C4	0.06 (17)	N2—C8—C9—C10	177.48 (10)
C1—N1—C5—C6	−179.64 (10)	C11—C9—C10—C11^i^	0.57 (17)
C3—C4—C5—N1	−0.30 (17)	C8—C9—C10—C11^i^	−179.67 (9)
C3—C4—C5—C6	179.39 (10)	C10—C9—C11—C10^i^	−0.57 (17)
N1—C5—C6—C7	−94.33 (12)	C8—C9—C11—C10^i^	179.68 (9)
C4—C5—C6—C7	85.97 (13)		

Symmetry codes: (i) −*x*, −*y*, −*z*.

### Hydrogen-bond geometry (Å, °)

**Table:**

Cg1 and Cg2 are the centroids of the C1–C5/N1 and C9–C11/C9'–C11' rings, respectively.

**Table:**

*D*—H···*A*	*D*—H	H···*A*	*D*···*A*	*D*—H···*A*
C3—H3···N1^ii^	0.95	2.74	3.544 (3)	143 (3)
C4—H4···N2^ii^	0.95	2.69	3.593 (2)	159 (4)
C7—H7A···N1^iii^	0.99	2.87	3.847 (2)	171 (5)
C2—H2···Cg1^iv^	0.95	2.88	3.826 (4)	172 (5)
C6—H6A···Cg2^v^	0.99	2.90	3.508 (3)	120 (2)

Symmetry codes: (ii) *x*, *y*, *z*+1; (iii) *x*−1, *y*, *z*; (iv) *x*+1/2, −*y*+1/2, *z*+1/2; (v) *x*+1, *y*, *z*+1.
